# Correlation of Clinical Characteristics of Meniere’s Disease and Its Patient-Oriented Severity Index (MD POSI)

**DOI:** 10.3390/audiolres15040099

**Published:** 2025-08-06

**Authors:** Josip Novaković, Ana Barišić, Erik Šuvak, Emili Dragaš, Petar Drviš, Tihana Mendeš, Jakov Ajduk, Siniša Maslovara, Andro Košec

**Affiliations:** 1 Department of Otorhinolaryngology, General Hospital Zabok, 49210 Bračak, Croatia; novakovicjosip@gmail.com; 2 Health Care Centre Zagreb West, 10110 Zagreb, Croatia; ana.barisic1007@gmail.com; 3 School of Medicine, University of Zagreb, 10000 Zagreb, Croatia; suvakerik277@gmail.com (E.Š.); emili.dragas1@gmail.com (E.D.); jakov.ajduk@gmail.com (J.A.); 4 Department of Otorhinolaryngology and Head and Neck Surgery, Clinical Hospital Sveti Duh, 10000 Zagreb, Croatia; pdrvis@gmail.com; 5 Department of Otorhinolaryngology and Maxillofacial Surgery, Faculty of Medicine, Josip Juraj Strossmayer University of Osijek, J. Huttlera 4, 31000 Osijek, Croatia; tihanamendes811@gmail.com; 6 Department of Otorhinolaryngology, Head and Neck Surgery University Hospital Centre Osijek, J. Huttlera 4, 31000 Osijek, Croatia; 7 Department of Otorhinolaryngology and Head and Neck Surgery, University Hospital Centre Sestre Milosrdnice, 10000 Zagreb, Croatia; 8 Department of Otolaryngology, National Memorial Hospital ‘‘Dr. Juraj Njavro’’ Vukovar, Zupanijska 35, 32000 Vukovar, Croatia; sinisamaslovara@yahoo.com

**Keywords:** Meniere’s disease, MDPOSI questionnaire, quality of life

## Abstract

**Background**: Meniere’s disease is characterized by a triad of vertigo episodes, fluctuating hearing loss, and tinnitus. The disease is followed by a loss of quality of life in patients, with the severity depending on the individual and the stage of the disease. Since there are no quantitatively validated tests that connect all elements of the disease, the only source of subjective data that can be analyzed is the disease diary and questionnaires, among which the MDPOSI (Meniere’s Disease Patient-Oriented Symptom-Severity Index) stands out as a designated quality-of-life assessment tool. This study aims to evaluate the differences in the questionnaire depending on the clinical characteristics of the disease. **Methods**: The study recruited 60 patients, with clinical variables including age, gender, disease laterality, caloric testing results, and PTA results, the presence of spontaneous nystagmus, pathological values of calorimetric testing, or rotatory chair testing abnormalities. **Results**: The appearance of spontaneous nystagmus showed a significant association with worse hearing threshold values at 500 Hz (*p* = 0.036, OR 4.416) and higher. Worse SRT scores correlated with Q1 (*p* = 0.011), Q2 (*p* = 0.028), Q4 (*p* = 0.045), Q5 (*p* = 0.013), and the total MDPOSI score (*p* = 0.008, 0.339). Multivariate analysis showed that a higher total value of the MDPOSI questionnaire was statistically significantly associated with older age (*p* = 0.042) and spontaneous nystagmus (*p* = 0.037). **Conclusions**: There is a correlation between the clinical characteristics of Meniere’s disease and the MDPOSI questionnaire, making it useful for assessing quality of life and disease progression.

## 1. Introduction

Meniere’s disease (MD) is a chronic illness characterized by a triad of symptoms (episodic vertigo, fluctuating hearing loss, and tinnitus). It affects around 50–500 people per 100,000 in the general population. Meniere’s disease is often accompanied by nausea, vomiting, a feeling of fullness and pressure in the affected ear, and nystagmus [1,2]. Women are more often affected than men, and Caucasians are the most affected ethnic group [3]. A genetic predisposition has been found in 2.6–12% of patients, with autosomal dominant inheritance with an incomplete penetrance as the possible mechanism [3]. In familial cases, more severe clinical symptoms were observed [3].

A pathohistological finding associated with Meniere’s disease is endolymphatic hydrops. Endolymphatic hydrops extends the endolymphatic space of the inner ear into the perilymphatic space, affecting both the vestibular and the cochlear spaces. The sacculus and cochlear duct are most often affected [1]. It most commonly affects one ear, but studies report on a wide range of bilaterally affected patients (2–75%). Today, with modern imaging techniques, it is revealed that there is a significant number of contralateral hydropic changes in the contralateral ear known as the asymptomatic contralateral ear [1,3,4,5,6]. The severity of the disease varies depending on the individual and the stage of the disease. In the acute phase, vestibular complaints predominate. Over time, the vertigo weakens, and the hearing loss progresses along with a feeling of unsteadiness [7]. In the symptomatic phase, which can last for decades, patients have a significant loss of quality of life that is not easy to interpret.

The goals of treatment of Meniere’s disease are to prevent or reduce the severity and frequency of vertigo attacks, to relieve or prevent hearing loss, aural fullness, and tinnitus, and to improve the quality of life [8]. The treatment models for Meniere’s disease are the conservative, noninvasive (sodium diet, histamine agonists and antagonists, diuretics), invasive models (intratympanic corticosteroid or gentamycin treatment and surgery), and lifestyle modifications (e.g., diet) [8]. In an effort to improve the patient’s daily activities and quality of life, some authors suggest treating vertigo with transtympanic tube placement, labyrinthectomy, or occlusion of the semicircular canals and hearing correction with cochlear implantation, which enables better resocialization if the patient returns to usual daily and business activities [8,9,10]. If the goal is to preserve hearing and reduce vertigo, the treatment options often include betahistine, diuretics such as acetazolamide or isosorbide, and corticosteroids either systemically or intratympanically [9,10].

The aforementioned treatments have their limitations; their efficacy and possible adverse effects should be taken into consideration [4,7,8]. Patients with Meniere’s disease have a severely impaired quality of life, including social and psychological aspects. Common comorbidities that arise from it are anxiety and/or depression, with 33% of men and 41% of women affected with MD carrying diagnoses of depression [8]. Because of this, they isolate themselves from other people and avoid or reduce all their daily activities [11,12]. Unfortunately, how to correctly measure the quality of life of patients with Meniere’s disease still remains unknown.

Since there are no quantitatively validated tests that connect all elements of the disease, the only source of subjective data that can be analyzed is the disease diary and questionnaires, among which the MDPOSI (Meniere’s Disease Patient-Oriented Symptom-Severity Index) stands out [13]. This questionnaire contains 16 questions about quality of life during the attack and between vertigo attacks in the areas of balance, hearing, memory, and daily activities. It is a reliable instrument to assess the impact of Meniere’s disease on patients’ quality of life [13,14,15]. We hypothesize that the changes in quality of life in MD patients estimated by this questionnaire correlate with objective clinical findings. The aim of our study was to evaluate the differences in the MDPOSI questionnaire depending on the stage and severity of Meniere’s disease.

## 2. Materials and Methods

**Figure A1:**
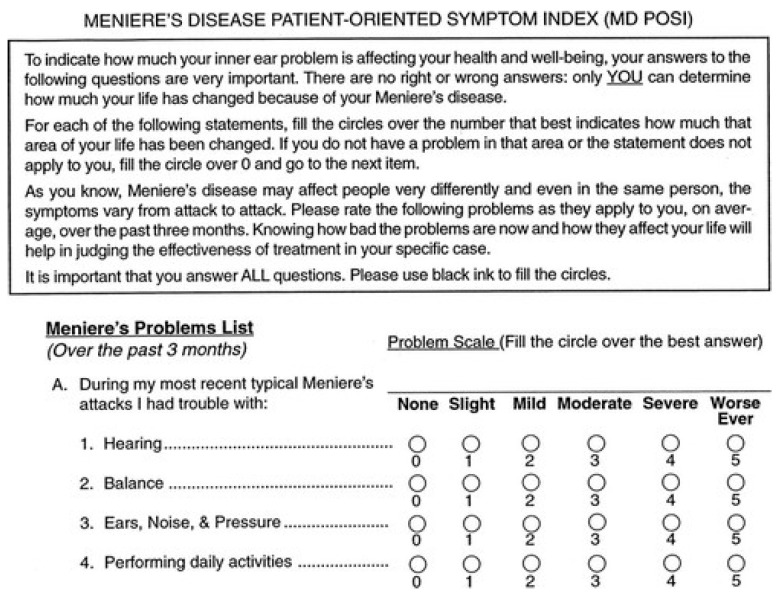
The first part of the Meniere’s disease patient-oriented symptom index (MD POSI).

**Figure A2:**
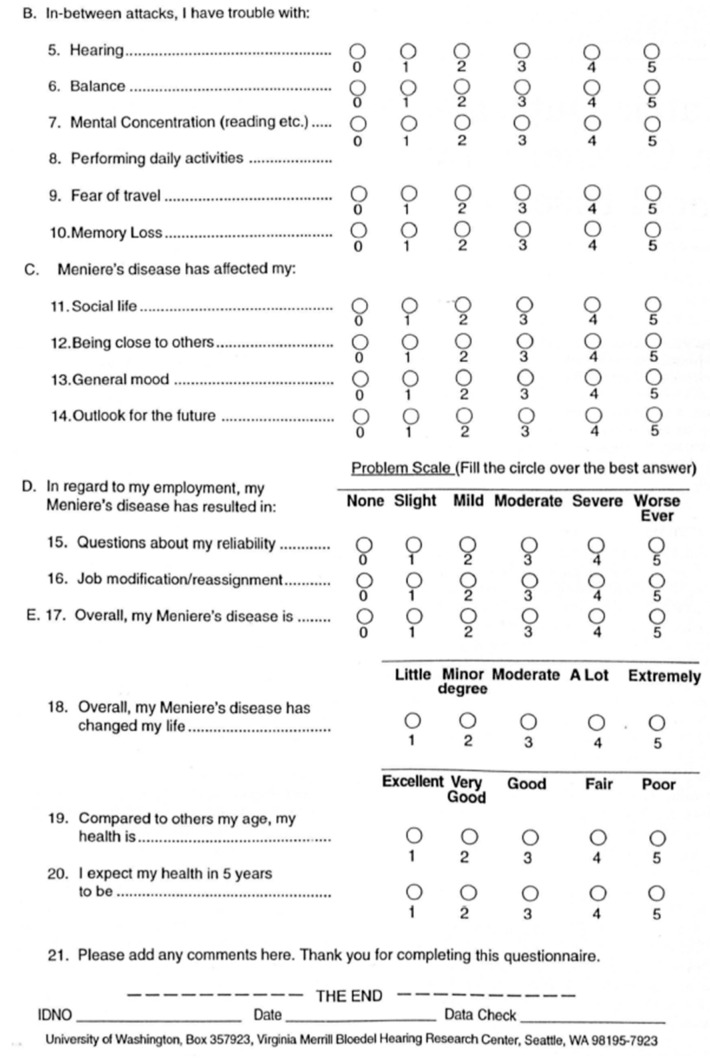
The second part of the Meniere’s disease patient-oriented symptom index (MD POSI).

This is a single-center, retrospective cohort observational study on patients with definitive Meniere’s disease in a tertiary audiology center. The results were obtained during 2021–2025 and assembled according to STROBE guidelines. The study protocol was approved by the Ethics Committee of University Hospital Sestre milosrdnice (EP 251-29-11-21-01-5). Written informed consent was obtained from all participants. Patients were asked to fill out the MDPOSI questionnaire (Figure A1 and Figure A2) and were informed about the study taking place.

### 2.1. Inclusion and Exclusion Criteria

Subjects were included if they had definite Meniere’s disease with co-existing conditions affecting hearing, balance, and the ability to fill out the questionnaires truthfully. The revised American Academy of Otolaryngology-Head and Neck Surgery (AAO-HNS) diagnostic criteria define “Definite MD” and “Probable MD” [4,9]. “Probable MD” consists of two or more spontaneous episodes of vertigo, each lasting 20 min to 24 h and fluctuating aural symptoms (hearing, tinnitus, fullness) in the affected ear [9]. “Definite MD” is diagnosed by additional audiometrically documented unilateral hearing loss, during or after one of the episodes of vertigo, lasting 20 min to 12 h [9]. The exclusion criteria removed patients who did not fit the criteria for definitive Meniere’s disease, had alternative diagnoses that could cause clinical symptoms, or did not give full informed consent.

### 2.2. Variables, Complementary Tests, and Descriptions

The clinical variables included age, gender, whether Meniere’s disease was unilateral or bilateral, caloric testing results and pure tone audiometry (PTA) results in dB on 250, 500, 1000, 2000, 4000 and 6000 Hz, presence of spontaneous nystagmus, pathological values of calorimetric testing (>30% of difference with respect to the contralateral response) or rotatory chair testing abnormalities. Speech intelligibility was evaluated through two parameters: the speech recognition threshold (SRT) and the maximum word recognition score, determined by delivering speech stimuli at an intensity corresponding to SRT, and adding up to 35 dB HL.

Subjects needed to complete all testing phases and provide full documentation and the questionnaires. Before study inclusion, all subjects underwent pure tone average (PTA), speech audiometry (WRS), and full videonystagmographic testing. This analysis did not include additional audiological tests, such as tympanometry, acoustic reflex testing, and otoacoustic emission.

### 2.3. Development and Content of the Questionnaire

The Meniere’s Disease Patient-Oriented Severity Index (MDPOSI) is a validated instrument with good inter-item consistency with 16 question domains regarding various aspects of Meniere’s disease attacks, showing a Cronbach’s alpha of 0.928, compiled by Gates et al. [13]. The MD POSI asks about symptoms during and between vertigo attacks over the preceding 3 months in the areas of balance, hearing, memory, and daily activities such as sleeping and driving. The effects of the disorder on the personal, social, and occupational aspects of their lives are also rated, alongside global questions about the effect of Meniere’s disease on their lives, overall health (now and in 5 years), and satisfaction with treatment.

### 2.4. Statistical Analysis

The values of the MDPOSI questionnaire were also included in the analysis, using the individual question values (Q1–Q16) and the total score. MDPOSI questionnaire values were correlated with clinical variables. In order to define further subgroups within the sample, related to the subjective and objective variables, we performed a two-step cluster analysis that included categorical and continuous variables. The two-step cluster analysis procedure is an exploratory tool designed to reveal natural groupings (or clusters) within a dataset that would otherwise not be apparent. The algorithm employed by this procedure has several desirable features that differentiate it from traditional clustering techniques. (A) It can handle categorical and continuous variables: by assuming variables to be independent, a joint multinomial–normal distribution can be placed on categorical and continuous variables. (B) Automatic selection of a number of clusters: by comparing the values of a model-choice criterion across different clustering solutions, the procedure can automatically determine the optimal number of clusters. We used log-likelihood as a distance measure between the clusters. We did not use feature engineering, and the cluster analysis was fed raw data. The clustering criterion was Schwartz’s Bayesian Criterion, and cluster cohesion was measured by the silhouette measure of cohesion and separation. We used the Chi-square test of independence to determine whether the variables were independent. Data distribution was calculated using the Kolmogorov–Smirnov test. Differences between clusters were analyzed using the Mann–Whitney U test for nonparametric variables.

Data distribution was calculated using the Kolmogorov–Smirnov test. Differences between clusters were analyzed using the binary logistic regression model, the ANOVA test, and Pearson correlation coefficient analysis. All tests of statistical significance were performed using a two-sided 5% type I error rate.

Statistical analysis was performed using SPSS (Version 22.0., 2013. IBM SPSS Statistics for Windows, Armonk, NY, USA: IBM Corp.) using standard descriptive statistics and frequency tabulation as indicated.

## 3. Results

There were 60 patients in the study, with 37 female and 23 male patients. The patient demographics and the summary of the MDPOSI, PTA, SRT, and VNG values are shown in Table 1.

**Figure 1:**
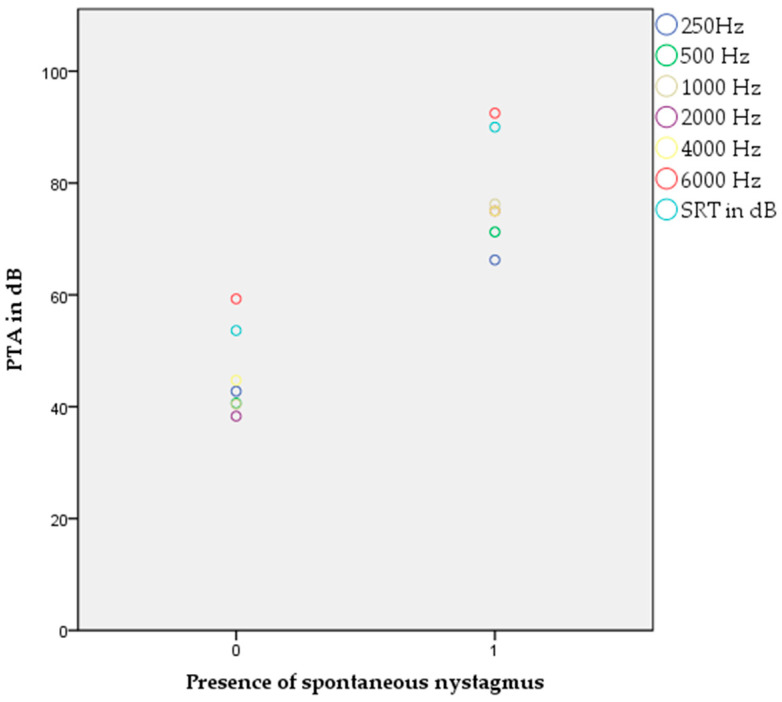
Appearance of spontaneous nystagmus and significant correlation to pure tone audiogram and SRT values (*p* < 0.05, binary logistic regression).

Binary logistic regression with the appearance of spontaneous nystagmus as a dependent variable showed a significant correlation with worse hearing threshold values at 500 Hz (*p* = 0.036, OR 4.416), 1000 Hz (*p* = 0.013, OR 6.166), 2000 Hz (*p* = 0.005, OR 7.806), 4000 Hz (*p* = 0.021, OR 5.532), 6000 Hz (*p* = 0.032, OR 4.622), and SRT values on the right side (*p* = 0.018, OR 5.580) (Figure 1).

When the occurrence of the pathological asymmetry in the calorimetric test was taken into the model as a dependent variable, the model showed a significant association with a worse hearing threshold at 2000 Hz (*p* = 0.042, OR 4.116), 4000 Hz (*p* = 0.012, OR 6.375), and 6000 Hz (*p* = 0.005, OR 7.842).

When the appearance of a pathological rotation test was taken into the model as a dependent variable, the model showed a significant correlation with questions Q1 (*p* = 0.011, OR6.436), Q4 (*p* = 0.023, OR 5.143) (hearing and daily activities during attack), and Q8 (*p* = 0.032, OR 4.572) (daily activities between attack).

Multivariate analysis (ANOVA) showed that a higher total value of the MDPOSI questionnaire was statistically significantly associated with older age (*p* = 0.042, F 1.962) and with the occurrence of spontaneous nystagmus (*p* = 0.037, F 2.010).

**Figure 2:**
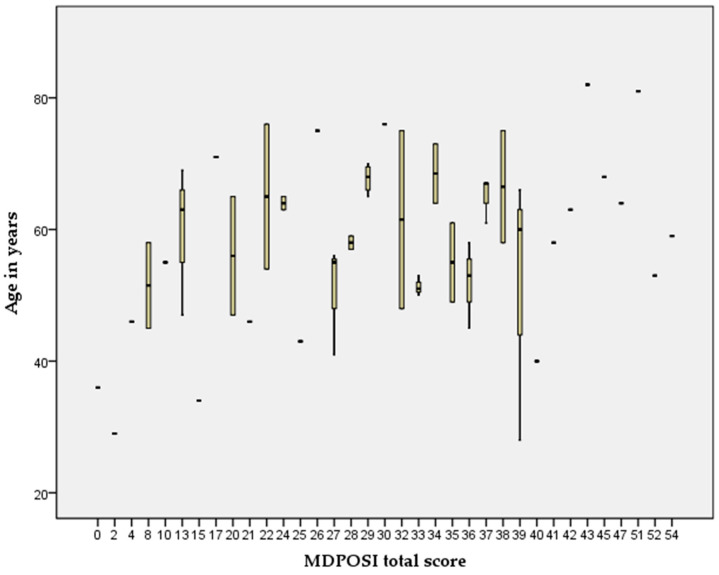
Association between higher MDPOSI questionnaire values and older age (*p* = 0.008, 0.342, Pearson’s correlation).

**Figure 3:**
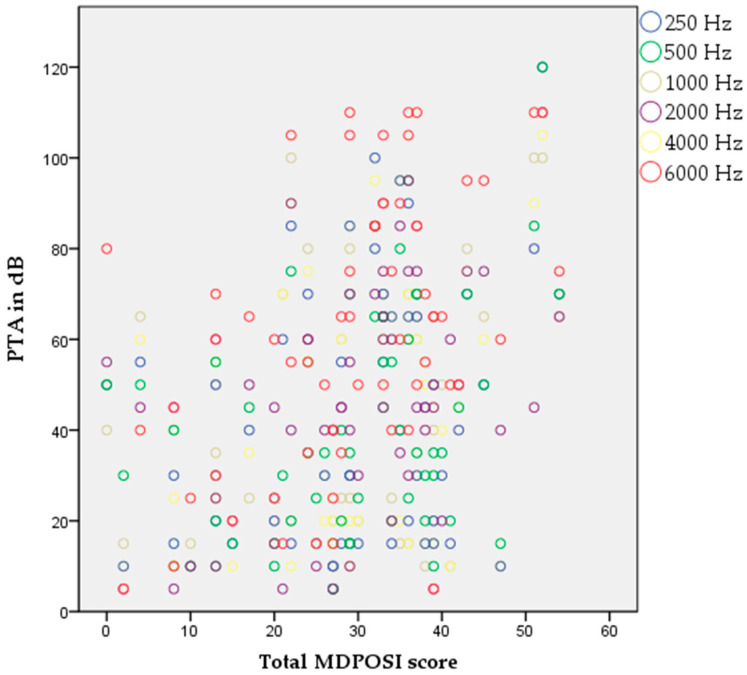
Association between higher MDPOSI questionnaire values and higher PTA values on all tested frequencies, 250 Hz-6000 Hz (*p* < 0.05, Pearson’s correlation).

**Figure 4:**
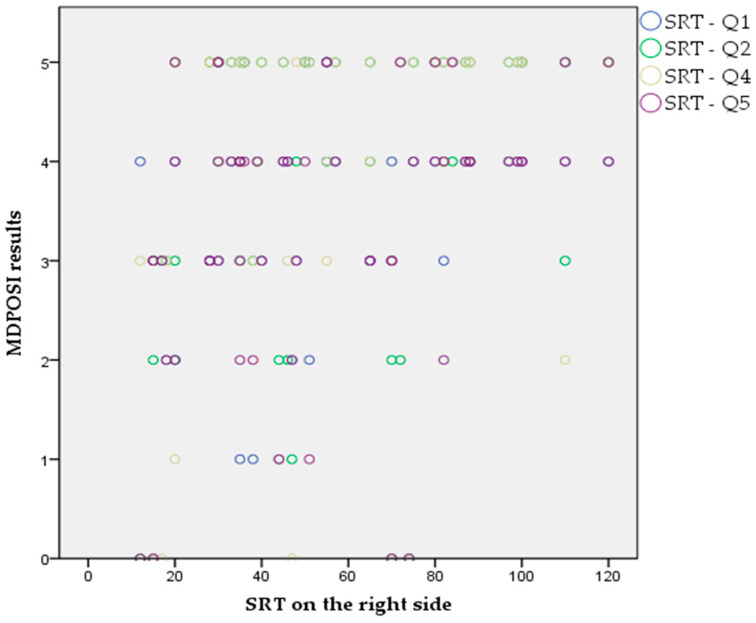
Association between Q1, Q2, Q4, and Q5 questions of the MDPOSI and SRT values on the right side (*p* < 0.05, Pearson’s correlation).

**Figure 5:**
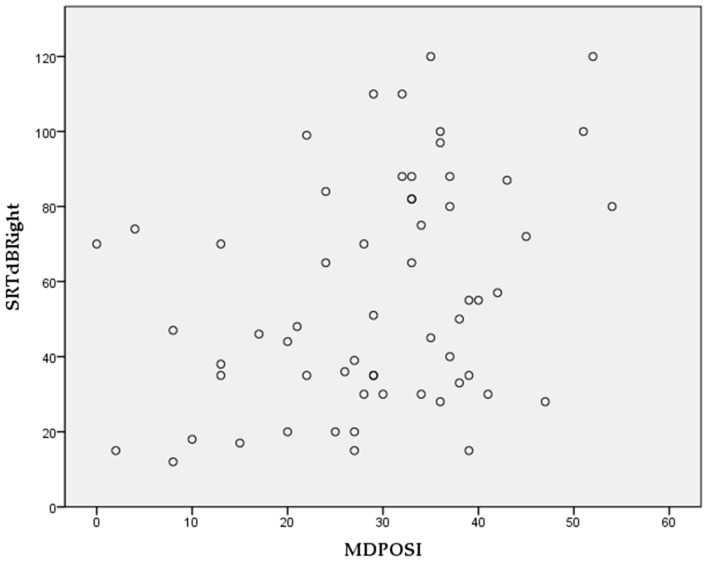
Association between total MDPOSI score and SRT values on the right side (*p* = 0.008, 0.339, Pearson’s correlation).

The Pearson’s correlation coefficient showed statistically significant associations between higher MDPOSI questionnaire values and older age (*p* = 0.008, 0.342), and worse hearing threshold at 250 Hz (*p* = 0.020, 0.300), 500 Hz (*p* = 0.016, 0.310), 1000 Hz (*p* = 0.011, 0.326), 2000 Hz (*p* = 0.004, 0.368), 4000 Hz (*p* = 0.001, 0.413), and 6000 Hz (*p* = 0.001, 0.461) (Figure 2 and Figure 3). When analyzing SRT values for the right ear, a significant correlation was shown with questions Q1 (*p* = 0.011, 0.325), Q2 (*p* = 0.028, 0.284), Q4 (*p* = 0.045, 0.260), Q5 (*p* = 0.013, 0.318), and the total MDPOSI score (*p* = 0.008, 0.339) (Figure 4 and Figure 5). This was confirmed in the left-ear SRT values, showing a significant correlation with question Q5 (*p* = 0.021, 0.298).

When analyzing the cluster distribution, the sample was separated into two significant clusters, with the first cluster consisting of 15 patients and the second consisting of 32 patients. The first cluster showed significantly better results compared to the second cluster regarding PTA values at 250 Hz (33.3 ± 14.6 dB vs. 67.8.3 ± 11.8 dB), 500 Hz (32.3 ± 17.6 dB vs. 65.5 ± 12.5 dB), 1000 Hz (27.7 ± 15.2 dB vs. 63.6 ± 14.2 dB), 2000 Hz (27 ± 15 dB vs. 56.6.3 ± 15.9 dB), 4000 Hz (36 ± 20.3 dB vs. 59.8 ± 21.2 dB), and 8000 Hz (47.3 ± 22.4 dB vs. 75.2 ± 24.9 dB) and a difference in caloric testing between the left and right sides (15.33 ± 24.7% vs. 26.13 ± 31.9%). Cluster two had a much higher prevalence of female patients (22 female vs. 8 male) (all Mann–Whitney U Test, *p* < 0.001).

## 4. Discussion

The aim of our study was to demonstrate the differences in the MDPOSI questionnaire scores depending on the stage and severity of Meniere’s disease and to show a correlation between the questionnaire and the clinical tests often performed to diagnose Meniere’s disease.

The results should be interpreted with regard to the pathophysiology of Menière’s disease and its primary mechanism of dysregulation of endolymphatic fluid homeostasis within the inner ear. It is widely hypothesized that endolymphatic hydrops play a central role in the disease process. Histopathological studies have demonstrated dilation of the scala media and cochlear duct in affected individuals, which correlates with clinical symptoms [15]. Additionally, immune-mediated processes and genetic predispositions have been implicated, suggesting a multifactorial etiology. Disruption of the endolymphatic space leads to the abnormal stimulation of hair cells and neural elements, resulting in the characteristic episodic vertigo and progressive hearing loss, making up the bulk of clinical symptoms [16].

In our analysis, we observed a significant correlation between worse hearing threshold values and the appearance of spontaneous nystagmus. Another clinical sign positively associated with worse hearing threshold at 2000 Hz, 4000 Hz, and 6000 Hz was the occurrence of pathological asymmetry in the calorimetric test. As both the presence of spontaneous nystagmus and calorimetric testing assess the vestibular function, the results infer a clinical correlation between significant hearing loss and the degree of vestibular dysfunction. This result is comparable with a study by McMullen K. P. et al., in which they observed a moderately significant correlation between caloric weakness and PTA, low PTA, and word recognition score (WRS) in the affected ear [17]. Moreover, a similar conclusion with a similar patient sample size as ours (79 participants) was drawn in a research paper by Wang HM et al. [17], where a positive correlation between the weakness in caloric response and clinical hearing level was identified. Other studies have shown inconsistent results regarding an association between these variables, depending on the point in time these objective tests were performed, in relation to the patient’s current disease activity [18]. According to McMullen K P et al., it would be ideal to have at least two sets of tests, to minimize the effect of symptom fluctuation and to better analyze the individual course of the disease [18].

When comparing the MDPOSI questionnaire results to different clinical tests, we found a statistically significant correlation between the appearance of a pathological rotation test and questions 1, 4, and 8. In question 1, the patients are asked to state the rate of hearing impairment in their latest Meniere attack, whereas in question 4, they are asked to evaluate to what degree their recent attack interfered with their daily activities. Question 8, on the other hand, evaluates time in between attacks and asks the patients to evaluate how much trouble they had in performing daily activities during that time. The decrease in everyday functioning was found both during the attacks and in between them, indicating that the vestibular portion of the Meniere’s triad is a burden to patients even in asymptomatic periods. This anxious anticipation of vertigo is a known phenomenon and has already been described in the literature [19]. A similar finding was noted in a study by Gates and Verrall [13]. The patients were divided into three groups, based on the perceived severity of their vertigo spells, and were given the MDPOSI questionnaire. In the end, they analyzed each individual item and their scores and found that the items that varied between the vertigo categories were Q4 and Q8, also indicating the impact of vertigo on everyday life and overall mental status and, consequently, quality of life [13]. One study found that, during MD attacks, the rating of the quality of well-being corresponds to the most debilitating conditions [8]. Vestibular impairment in Meniere’s disease significantly impacts individuals’ everyday life, affecting their balance, spatial orientation, and overall mobility. Patients often experience symptoms such as dizziness, postural instability, and oscillopsia, which can lead to difficulties in performing routine activities like walking, reading, and driving. These impairments can cause increased risk of falls, injury, and reduced independence, thereby impairing quality of life [14].

Many studies indicate that vestibular dysfunction is associated with persistent gait disturbances and difficulties with dual-task activities that require cognitive and motor coordination [15]. Patients frequently report limitations in social participation and work-related tasks, contributing to psychological effects such as anxiety and depression [16]. The severity of functional impairment correlates with the extent of vestibular damage, but even mild impairments can significantly affect daily functioning [17].

Rehabilitation strategies, including vestibular therapy and balance training, aim to mitigate these impacts and improve patients’ capacity to perform everyday activities. However, the heterogeneity in impairment levels necessitates tailored approaches for effective management, where instruments like MDPOSI may especially be useful [20,21]. Understanding the scope of daily life impairment underscores the importance of early diagnosis and comprehensive rehabilitative interventions to enhance the quality of life of patients with vestibular disorders.

As we demonstrated before, there is a positive association of pathological asymmetry in the calorimetric test with worse hearing threshold at higher frequencies and SRT scores in the affected ear, with questions regarding trouble with hearing, balance, ears, nose, and pressure during the attack and trouble with hearing in-between attacks, as well as the total MDPOSI score. SRT values demonstrate a known phenomenon—functional audiometric dissociation in unilateral and bilateral Ménière’s disease, where affected ears show poorer speech recognition and require higher intensities despite similar PTA values [19].

It is worth noting that, although hearing loss in MD affects the lower frequencies most often, due to the involvement of the apical cochlea, the correlation of impaired SRT scores with disease progression makes this finding interesting, suggesting that hearing loss progression may also closely follow overall disease progression. This may be due to the average age of the participants (60 years), as presbycusis is highly prevalent in ages above 50 [18,19,20], but cannot be attributed solely to advancing age. Along with the rotatory chair test, caloric testing also assesses the integrity of the horizontal canal, indicating the possible comparison of these two findings. Murphy and Gates found that hearing loss both during and between attacks was rated as the most severe MD symptom, but they did not find a correlation with patient-perceived severity of vestibular symptoms, as rated by the MDPOSI questionnaire [22,23]. Their explanation is that hearing loss is a more fixed symptom, while in contrast, there is a greater fluctuation in the vestibular symptoms.

Lastly, we have also found a statistically significant association between a higher total value of the MDPOSI questionnaire and older age, as well as overall worse hearing in all tested frequencies, and the occurrence of spontaneous nystagmus. The latter two come as no surprise, since the overall higher score of the MDPOSI questionnaire indicates a worse perception of the status of the disorder. However, the connection between old age and a higher total value poses a question as to whether symptoms really do stabilize with time. One study concluded that, in elderly patients or in patients with long-standing MD, there may not be typical MD-like temporal patterns, but episodes that manifest more like severe imbalance or ‘‘vague’’ dizziness [8,22]. Since we did not include disease duration as a variable in our research, we cannot correlate old age with a longer course of the disease.

It is well known that Meniere’s disease does not have reliable predictors of prognosis. The symptoms of Meniere’s disease may stabilize, but in an unknown time frame. The main problem regarding the quality of life and the main reason for seeking physicians’ help is vertigo [12]. During exacerbations of the disease and between them, apart from the measurement of spontaneous or pathological nystagmus and the tone audiogram, there are no measuring instruments that could be used to measure patients’ quality of life [13]. The absence of an objective measure makes it difficult to estimate the severity of symptoms and the patient’s condition [23,24]. Therefore, we have to rely on subjective data provided by patients. This data depends on the current condition and mood of the patient [1]. The MDPOSI questionnaire is a standardized, validated questionnaire that enables the analysis and comparison of data obtained by surveying patients and comparison with objective tests [13,20]. A higher value of the answers obtained by the questionnaire is significantly related to the progression of the disease. While most studies rely on subjective data collected directly from the patient, data obtained from this questionnaire make it suitable for assessing the symptoms of vertigo and hearing loss [9].

This study, however, has potential limitations. First, we did not take into consideration any comorbidities of the patients that could greatly impact their answers in the questionnaire, such as other chronic illnesses that directly impact balance or overall quality of life. Second, most similar studies consider the duration of the disease, which we did not include as one of the variables. Therefore, it was somewhat harder to compare results and draw conclusions.

## 5. Conclusions

This study showed correlations between the MDPOSI questionnaire and the clinimetrics of Meniere’s disease, regarding the everyday functioning of patients and hearing impairment during attacks. A correlation between hearing loss and the degree of vestibular dysfunction (spontaneous nystagmus and asymmetric calorimetric testing) in MD patients was also found. The MDPOSI questionnaire could represent a valuable tool in estimating Meniere’s disease progression.

## Figures and Tables

**Table 1:** Summary of clinical and MDPOSI data.

All Participants	n = 60
Male	n = 23
Female	n = 37
Right-sided MD	n = 34
Left-sided MD	n = 24
Bilateral MD	n = 2
Age in years	
Average ± SD	58.05 ± 12.31
PTA values in dB	
Right 250 Hz	44.3 ± 27.4
Right 500 Hz	42.7 ± 28.2
Right 1000 Hz	42.8 ± 27.9
Right 2000 Hz	40.8 ± 25.4
Right 4000 Hz	46.8 ± 25.3
Right 6000 Hz	61.5 ± 29.9
Left 250 Hz	36.3 ± 27.2
Left 500 Hz	35 ± 28.1
Left 1000 Hz	34.4 ± 26.8
Left 2000 Hz	33.2 ± 26
Left 4000 Hz	41.8 ± 27.1
Left 6000 Hz	56 ± 29.3
Speech recognition threshold	
Right (dB)	56.1 ± 29.5
Left (dB)	46.9 ± 29.7
Spontaneous nystagmus (N)	4
Pathologic caloric testing (N)	16
Pathologic rotatory chair test (N)	1
MDPOSI questions scores specific to the attack within 3 months	
Q1 (During my most recent typical Meniere’s attacks, I had trouble with hearing)	3.45 ± 1.37
Q2 (During my most recent typical Meniere’s attacks, I had trouble with balance)	3.87 ± 1.49
Q3 (During my most recent typical Meniere’s attacks, I had trouble with ears, nose, and pressure)	2.53 ± 1.79
Q4 (During my most recent typical Meniere’s attacks, I had trouble with performing daily activities)	3.83 ± 1.7
Q5 (In between attacks, I have trouble with hearing)	3.38 ± 1.33
Q6 (In between attacks, I have trouble with balance)	1.03 ± 1.2
Q7 (In between attacks, I have trouble with mental concentration)	0.48 ± 0.85
Q8 (In between attacks, I have trouble with performing daily activities)	0.7 ± 1.08
Q9 (In between attacks, I have trouble with the fear of travel)	0.92 ± 1.34
Q10 (In between attacks, I have trouble with memory loss)	0.47 ± 0.84
Q11 (Meniere’s disease has affected my social life)	1.73 ± 1.58
Q12 (Meniere’s disease has affected being close to others)	1.57 ± 1.56
Q13 (Meniere’s disease has affected my general mood)	2.17 ± 1.37
Q14 (Meniere’s disease has affected my outlook for the future)	1.77 ± 1.47
Q15 (In regard to my employment, my Meniere’s disease has resulted in questions about my reliability)	0.71 ± 1.21
Q16 (In regard to my employment, my Meniere’s disease has resulted in job modification/reassignment)	0.58 ± 1.099
Total score	29.18 ± 12.11

## Data Availability

Dataset is available upon request from the authors.

## Abbreviations

The following abbreviations are used in this manuscript:

MD	Meniere’s Disease
AAO-HNS	American Academy of Otolaryngology-Head and Neck Surgery
MDPOSI	Meniere’s Disease Patient-Oriented Symptom-Severity Index
PTA	Pure Tone Audiometry
WRS	Word Recognition Score
VOR	Vestibuloocular reflex
